# A critical review of microplastics in aquatic ecosystems: Degradation mechanisms and removing strategies

**DOI:** 10.1016/j.ese.2024.100427

**Published:** 2024-04-25

**Authors:** Sameh S. Ali, Tamer Elsamahy, Rania Al-Tohamy, Jianzhong Sun

**Affiliations:** a Biofuels Institute, School of the Environment and Safety Engineering, Jiangsu University, Zhenjiang, 212013, China; b Botany Department, Faculty of Science, Tanta University, Tanta, 31527, Egypt

**Keywords:** Persistent organic pollutants, Bioremediation, Nanomaterial-enabled strategies, Aquatic environment, Ecotoxicity of microplastics

## Abstract

Plastic waste discarded into aquatic environments gradually degrades into smaller fragments, known as microplastics (MPs), which range in size from 0.05 to 5 mm. The ubiquity of MPs poses a significant threat to aquatic ecosystems and, by extension, human health, as these particles are ingested by various marine organisms including zooplankton, crustaceans, and fish, eventually entering the human food chain. This contamination threatens the entire ecological balance, encompassing food safety and the health of aquatic systems. Consequently, developing effective MP removal technologies has emerged as a critical area of research. Here, we summarize the mechanisms and recently reported strategies for removing MPs from aquatic ecosystems. Strategies combining physical and chemical pretreatments with microbial degradation have shown promise in decomposing MPs. Microorganisms such as bacteria, fungi, algae, and specific enzymes are being leveraged in MP remediation efforts. Recent advancements have focused on innovative methods such as membrane bioreactors, synthetic biology, organosilane-based techniques, biofilm-mediated remediation, and nanomaterial-enabled strategies, with nano-enabled technologies demonstrating substantial potential to enhance MP removal efficiency. This review aims to stimulate further innovation in effective MP removal methods, promoting environmental and social well-being.

## Highlights

• The implications of microplastics (MPs) are critical to aquatic ecosystems.
• Biological remediation is a potential approach for MP removal.
• The ecotoxicity and transport of MPs are reviewed.
• Advanced techniques for efficient MP removal are discussed.
• Nano-enabled technology for MPs exhibits significantly high efficiency.

## Graphical abstract

**Figure:**
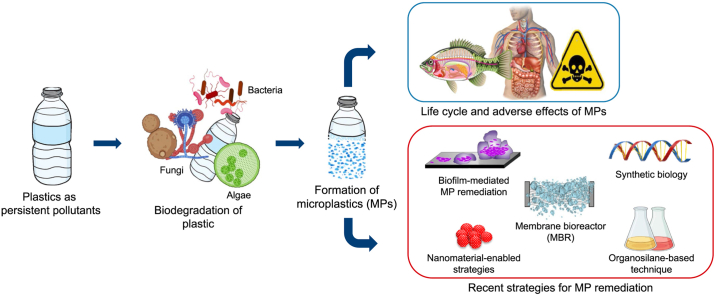

## 1 Introduction

Plastics and microplastics (MPs) are persistent pollutants increasingly becoming prevalent in the littering of all environmental niches worldwide [[1], [2], [3]]. The enduring characteristics and detrimental effects of plastic waste (PW) on aquatic organisms and humans have caused it a significant issue [4,5]. PWs account for over 75% of marine contaminants [6]. This is primarily due to the inflexible and non-biodegradable characteristics of plastic. Despite being regarded as a crucial resource for human existence, the Mediterranean Sea region has regrettably transformed into one of the most severely contaminated areas with plastics and MPs [7]. By 2025, it is projected that almost 11,000 million tons of PW might accumulate and be deposited in terrestrial and aquatic environments [8]. However, due to various biotic and abiotic processes, the current percentage of PW is below 5%. Moreover, the remaining PW is prone to deterioration, resulting in the formation of MPs [9].

MPs represent a class of refractory pollutants whose ubiquitous existence raises global concern. Consequently, these substances can damage various life forms and aquatic (including freshwater and marine), terrestrial, and remote arctic ecosystems [10]. MPs are utilized extensively in various fields due to their cost-effectiveness and high durability [11,12]. Substantial environmental and human health repercussions have been associated with MPs, defined as plastic particles smaller than 5 mm. Thompson et al. [13], who investigated oceanic plastic contamination in the United Kingdom, introduced the term “microplastics” exactly nineteen years ago. Subsequently, the scientific community and governmental organizations have displayed considerable interest in MPs. Plastics, introduced to the market in the latter half of the previous century, have caused considerable environmental threats due to their overproduction and widespread application across industries and products [1,4].

MPs are isolated or coexisting in the ecosystem with other components, including heavy metals, dyes, and persistent organic pollutants [[14], [15], [16]]. The MPs consumed by aquatic organisms are presented in Table 1 [[17], [18], [19], [20], [21], [22], [23], [24]]. The risks linked to the significant volumes of MPs released into aquatic ecosystems can be classified into physical damage and chemical toxicity [25]. The ingestion of MPs, whether through direct or indirect ingestion or accumulation in the food chain, poses a substantial risk to many organisms, including humans [3,5]. Thousands of MP debris are ingested due to the MPs escaping from the drinking water treatment facility and contaminating the tap water [26]. The ingestion of MPs harms various vital metabolism processes [27]. The interaction between MPs and other contaminants has been found to cause secondary pollution and toxicity in biota through ingestion [1,4].

**Table 1:** Effects of aquatic organisms on the ingestion of various types of microplastics.

Organism	Effect	Plastic type	Reference
*Centriscus cristatus*	Feeding decreases	PS	[17]
*Mytilus edulis*	Granulocytoma formulation	PE	[18]
*Calanus helgolandicus*	Reproduction decreases	PS	[19]
*Mytilus edulis*	Aggregation in soft tissues	PE	[20]
*Ostrea edulis*	Metabolic rate increases	PLA	[21]
*Balaenoptera physalus*	Toxicity symptoms increases	PP	[22]
*Arenicola marina*	Respiratory rate exaltation	PLA	[23]
*Allorchestes compressa*	Granulocytoma formulation	PS	[18]
*Artemia nauplii*	Liver swelling and aggregation	PE	[24]

Abbreviations: PS, polystyrene; PE, polyethylene; PLA, polylactic acid; PP, polypropylene.

As a matter of international concern, it is critical to develop effective strategies to address the challenges posed by MP pollution. Considerable endeavors have been expended, encompassing reducing materials, expanding recycling capabilities, and advancing bio-based feedstock. In addition, several conventions and regulations have been enforced to tackle concerns related to MPs. To mitigate MP pollution from water sources and regulate the discharge of MPs into waterways, the European Union has implemented a comprehensive plastic strategy [28]. Identifying the full spectrum of MP pollutants, including their morphology and surface chemistry, provides the first challenge in removing them from the environment. The identification and characterization of MP in environmental matrices have been established to produce trustworthy data on the toxicity of this pollutant [29]. This is established to facilitate the development of effective strategies for mitigating the effects of MP pollutants in aquatic systems. To date, various removal technologies have been implemented to mitigate the adverse effects of MPs [30,31]. Water treatment is an effective technique for decreasing the prevalence of MPs in various water systems, such as natural streams and industrial effluent, while also controlling the release of MPs at their origin. Despite the ability of numerous treatment technologies in water treatment facilities to remove considerable volumes of MPs, there remains potential for future efficiency improvement and overcoming these technologies' limitations.

Extensive technological innovations have occurred over the past five years, with the continuous exploration of new materials and techniques. Bioremediation appears to be an attractive strategy for mitigating the spread and effects of MPs. Nanotechnology-based techniques for eliminating pollutants have emerged as viable solutions to tackle the existing difficulties, either by enhancing efficiency or targeting untapped market segments [[32], [33], [34]]. In managing MPs, nano-enabled technology shows great potential for significantly improving the efficiency of MP removal, as well as their monitoring and identification, and for lowering the detectable threshold. Advanced nanomaterials, meticulously created and manufactured, have the necessary properties to eradicate MPs efficiently.

To this end, this review expands upon previous reviews by exploring the various aspects of MPs in aquatic systems, including their formation and ecotoxicological effects. This review emphasizes notable research gaps and gathers recent technological developments in removing MPs from aquatic environments. It also assesses various management strategies and treatment approaches for MP removal. Furthermore, it analyses the prospects of research in the pertinent domain and offers recommendations for mitigating the problem of MP pollution in aquatic systems. Hence, in-depth knowledge on MPs is obtained, which will eventually help in arousing awareness among the masses. To promote environmental and social well-being, it is anticipated that this review could spark additional concepts for implementing efficient strategies for MP removal.

## 2 Bibliometric analysis

**Fig. 1:**
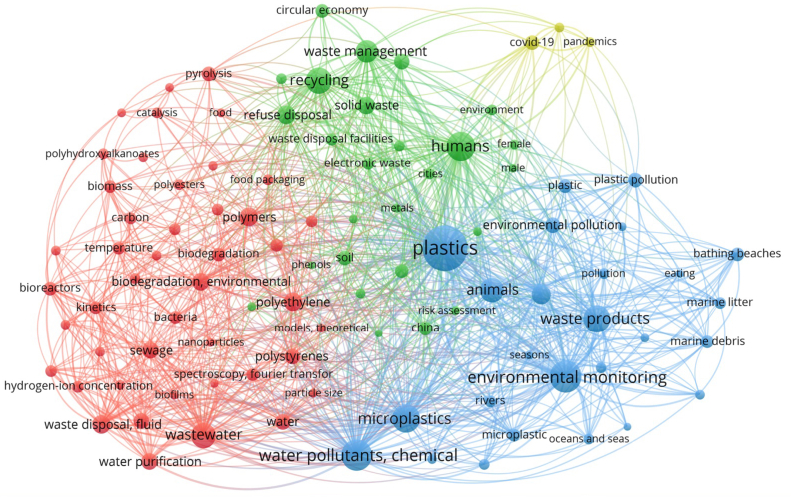
Network visualization map displaying the citation networks of the most frequently used keywords clustered together.

Bibliometric analysis aids in the determination of future research directions by providing a comprehensive overview and classification of past and present research. Thousands of documents can be analyzed concurrently with the help of mathematical statistics tools, enabling an efficient comprehension of research hotspots. Bibliometric analysis is an element of scientometrics that scrutinizes scientific activity within a particular research domain using mathematical and statistical methods [35]. The bibliometric study was conducted using the VOSviewer software, enabling the expansion and retrieval of information inside each cluster (Fig. 1). The network of terms is visualized using colors and nodes, where larger sizes represent descriptors with stronger links and the same color, whereas distant groups represent descriptors with fewer connections. The lines represent the connections between the groups. Upon examining the interwoven network of keywords, it is evident that certain terms were recurrently mentioned.

Bibliometric analysis is an advanced methodology employed by scholars worldwide to understand the dynamic patterns and trends that emerge within particular domains of knowledge. The bibliographic data for this study was subjected to bibliometric analysis using VOSviewer software (version 1.6.17). Four steps constitute the methodology for this review paper study: data identification and extraction, data screening, eligibility analysis, and bibliometric analysis. Notably, research focused on removing MPs is relatively new, initiating in 2014. In 2014 and 2015, there was a notable dearth of publications pertaining to the elimination of MPs, with only one publication each year. This number has significantly increased recently, reaching 145 in 2020. The surge in scientific inquiry may be ascribed to a confluence of elements, encompassing the flexibility granted to researchers as a result of lockdowns for coronavirus disease 2019 (COVID-19) and an expanding scientific preoccupation with effectively resolving the MPs dilemma and conforming to worldwide endeavors to reduce PW. A total of 201 research papers, obtained from the Scopus and Web of Science databases, covering the period from 2013 to 2023, were limited to articles written in English. The selected 100 keywords were divided into four clusters of 45, 26, 26, and 3 items in VOSviewer. The search was performed utilizing a combination of the following keywords: “animals”, “waste product”, “microplastics”, “water pollutants”, “rivers”, and “ocean and seas”, “polyethylene”, “biodegradation”, “polyprpylene”, “wastewater”, “water purification”, and “bacteria”, “humans”, “waste management”, “solid waste”, “recycling”, “heavy metals”, “risk assessment”, “China”, and “circular economy”, “covid-19” “pandemics”, and “sars-cov-2”. The descriptors were chosen via a survey that included network analysis of keyword co-occurrence using the complete counting approach (Fig. 1).

## 3 Biodegradation of plastic

**Fig. 2:**
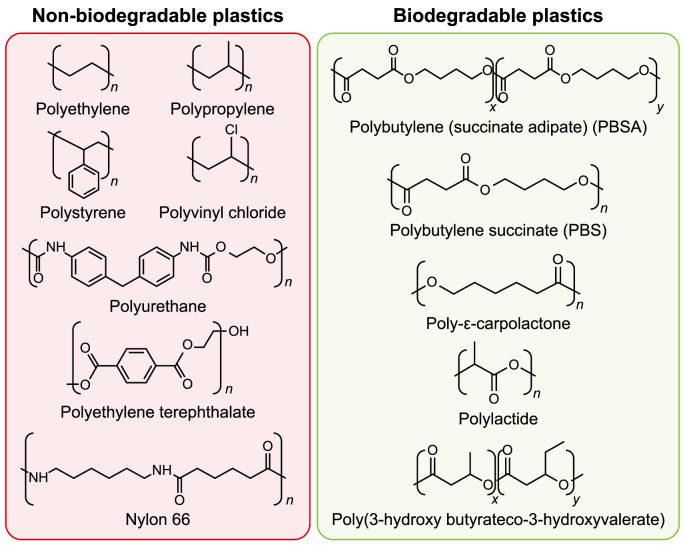
Different types of non-biodegradable and biodegradable plastic.

**Fig. 3:**
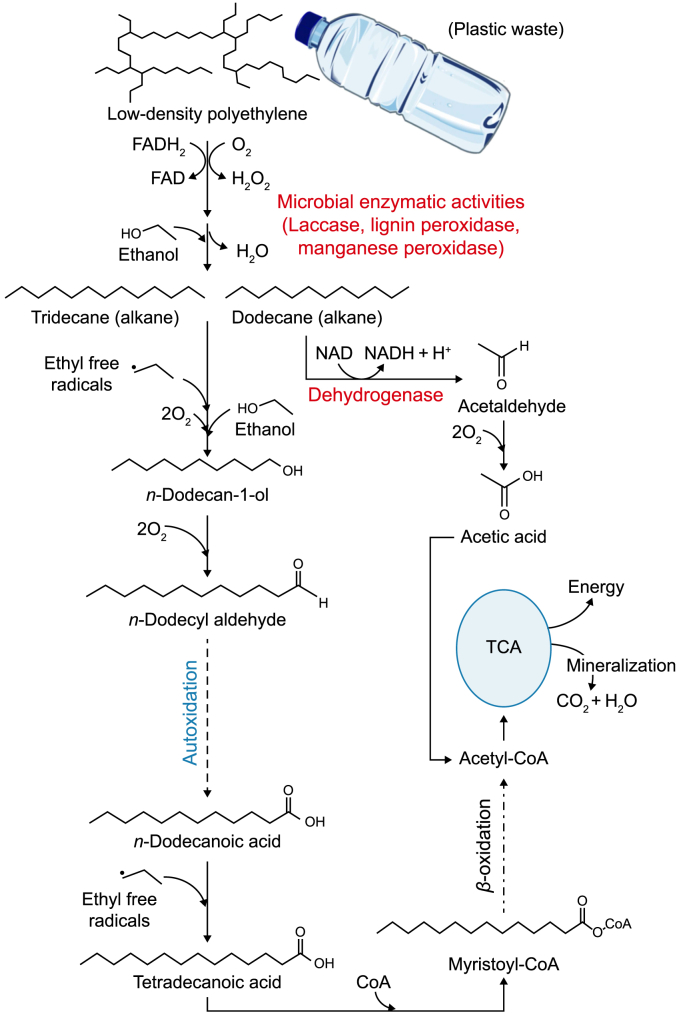
Microbial pathways for the biodegradation of polyethylene.

Plastics can be categorized into two main types: fossil- and bio-based plastics. Bio-based polymers can be categorized as either non-biodegradable or biodegradable (Fig. 2). Plastics contain a multitude of noteworthy chemical bonds, including ester bonds (-COO) in polyethylene terephthalate (PET), amide bonds (NH–CO) in nylon, and carbon backbone (C–C) compounds found in polyethylene (PE) and polypropylene (PP). Consequently, hydrolyzable ester linkages in polymers affect their biodegradability and the rate at which they decompose [36]. Monomers derived from petroleum sources are a common method of producing plastics [1,37,38]. It has been asserted that fossil-derived plastics, including PE, PET, PP, polystyrene (PS), polyvinyl chloride (PVC), and polyurethane (PUR), exhibit non-biodegradable properties [39]. Nevertheless, these polymers can undergo fragmentation due to physicochemical conditions, leading to MPs formation. Fig. 3 depicts the biodegradation of plastic waste, such as low-density polyethylene (LDPE), resulting in CO_2_, H_2_O, and energy production.

### 3.1 Bacterial-based plastic degradation

The ability to produce extracellular polysaccharides and establish biofilms on plastic surfaces was observed in various bacterial taxa [40]. Plastic degradation during wastewater treatment (WWT) has been extensively documented in many bacterial taxa, such as *Vibrio*, *Campylobacter*, and *Arcobacter* [41]. Bacteria possess the capability to effectively degrade MPs found in wastewater using bacterial enzymes, including PET hydrolase (PETase) and mono-(2-hydroxyethyl) terephthalate hydrolase (MHETase). This is attributed to the large surface-to-volume ratio of these particles, which provides an increased surface area for bacterial colonization and subsequent degradation.

*Stenotrophomonas panacihumi* exhibited the capability to enzymatically breakdown PP into both low- and high-molecular-weight fractions over 90 days [42]. In addition, *Aneurinibacillus* spp. and *Brevibacillus* spp. have the capacity to degrade PE and PP wastes. This degradation process was observed after a 140-day incubation period, resulting in a weight loss ranging from 37.2 to 45.7 [43]. Furthermore, *Pantoea* sp. and *Enterobacter* sp. exhibited the ability to decrease the weight of LDPE strips and pellets by 81% and 38%, respectively, following a 120-day incubation period [43]. *Bacillus amyloliquefaciens* BSM-1 and *B. amyloliquefaciens* BSM-2 were found to break down PW, which were then used to break down LDPE [44]. According to the findings of Kida et al. [45], the rate of biodegradation of PW can be determined by quantifying the CH_4_ and CO_2_ emissions that occur during the anaerobic digestion and mineralization of PW processes, respectively (Fig. 3). It is well-established that the incorporation of organic compounds and the presence of heteroatoms (e.g., nitrogen, oxygen, and sulphur) in PW enhance enzymatic and hydrolytic activity [46]. Biofilm formation by *Erythrobacter* sp. and *Alcanivorax borkum* has been implicated in the biodegradation of LDPE and its subsequent removal from aquatic ecosystems [47].

### 3.2 Fungal-based plastic degradation

The presence of considerable amounts of PW in filtered and polluted water sources, such as streams, rain, surface water, and oceans, has been documented [48]. A group of fungi, including *Fusarium* sp., *Aspergillus japonicas*, and *A. flavus*, could reduce the weight of LDPE by 30–36% when used as a carbon source [49]. This reduction was facilitated by the activity of microbial enzymes, as illustrated in Fig. 3. Various enzymes, including cutinases, lipases, and esterases, can catalyze the polyethylene adipate and polycaprolactone hydrolysis processes [50]. Numerous fungal species, including *Achromobacter* sp., *Rhizopus arrhizus*, *R. delemar*, and *Candida cylindracea*, have been recognized as important suppliers of lipases and esters, which are crucial for the biodegradation of PW [51]. On the other hand, a variety of metabolites, such as alcohol, alkanes, fatty acids, and aldehydes, were detected throughout the biodegradation process of LDPE by a yeast consortium (*Meyerozyma caribbica*, *M. guilliermondii*, and *Sterigmatomyces halophilus*) isolated from the gut of termites [12]. Lignin-degrading enzymes, including laccases and peroxidases, can degrade LDPE and generate CH_4_, CO_2_, and H_2_O using anaerobic and aerobic processes (Fig. 3) [11].

### 3.3 Algal-based plastic degradation

There is a scarcity of research examining the potential of various phytoplankton species to biodegrade PW and their contribution to reducing plastic pollution. Table 2 presents different algae species for plastic biodegradation [[52], [53], [54], [55], [56], [57], [58]]. The proliferation of phytoplankton on the surface of PW was promoted by the presence of essential elements such as water, sunlight, and nutrients [[59], [60], [61]]. Microalgae are a promising candidate for various applications due to their desirable characteristics. Microalgae can respire photoautotrophically without requiring organic carbon sources, in contrast to bacterial and fungal strains that harbor endotoxins [62]. This characteristic sets microalgae apart from the aforementioned biological pollutants, rendering them a desirable option for additional applications. Many non-toxic algal species can establish biofilms on plastic surfaces and inhabit polluted aquatic environments, such as ponds and lakes [63]. The process by which phytoplankton adhere to a plastic surface is facilitated by exopolysaccharide synthesis [64]. Enzymes, including lipases, esterases, and cellulases, can establish an interface with the plastic surface, thereby instigating PW's biodegradation and fragmentation process.

**Table 2:** Efficiency of algae in plastic degradation.

Polymer type	Algal species	Degradation time (day)	Reference
PE	*Scenedesmus dimorphus*, *Navicula pupula*, and *Anabaena spiroides*	45	[52]
PET	*Phaeodactylum tricornutum*	7	[53]
PE	*Oscillatoria subbrevis* and *Phormidium lucidum*	42	[54]
PE	*Nostoc carneum*	42	[55]
PET	*Spirulina* sp.	112	[56]
PE	*Uronema africanum*	30	[57]
PP	*Spirulina* sp.	112	[56]
PE	*Phormidium tenue, Oscillatoria tenuis, Monoraphidium contortum, Microcystis aeruginosa, Closterium constatum, Chlorella vulgaris,* and *Amphora ovalis*	ND	[58]

Abbreviations: PE, polyethylene; PP, polypropylene; PET, polyethylene terephthalate; ND, not detected.

Microalgae can flourish on PET surfaces and produce PETase, an extracellular enzyme vital to biodegradation [65]. Therefore, incorporating algae into the PW-containing solution significantly enhances the biodegradation process. As a result, the enzymatic activity of phytoplankton appears to accelerate the decomposition process significantly [66]. Microalgae can undergo genetic transformation, producing microbial cells that can proliferate and produce polymer-degrading enzymes. A genetic modification was executed on *Chlamydomonas reinhardtii* to produce PETase, which was then evaluated in connection with PET [67]. Surface degradation is indicated by the existence of holes and cavities on the plastic surface [53]. Therefore, synthetic biology provides a feasible approach to demonstrate ecologically sound methodologies for the degradation of PW via microalgae. Typically, the biodegradation of PW is accelerated by the production and secretion of specialized enzymes that degrade the intricate polymeric structure into more manageable components. Nevertheless, further investigation is necessary to understand the underlying mechanisms and improve this approach for practical applications in PW management.

## 4 Mechanisms of plastic degradation

Once generated, MPs are conveyed through wastewater discharge before entering freshwater or marine systems or municipal effluents [68,69]. Because of their diminutive dimensions, these particulates evade traditional water filtration systems and enter aquatic environments, where they may pose a risk to aquatic and terrestrial organisms. Secondary MPs result from the environmental stress, cracking, and degradation caused by biological and ultraviolet (UV) attacks, which reduce larger plastic pieces to smaller fragments [1,4]. The degradation of plastics is also facilitated by the abrasive impact of waves striking the coastal sand and rocks [70]. Abiotic degradation pertains to converting polymers into MPs via the synergistic influence of chemical and physical agents. The major factors contributing to the abiotic degradation of plastics are UV attacks, oxidation, thermal impacts, hydrolysis, and abrasion due to wave action in the seas and oceans [6,71]. Plastics typically undergo biodegradation in terrestrial and aquatic environments. Particularly at the benthic level, biodegradation rates are negligible in aquatic environments. This is predominantly due to the reduced microbial population in these environments. Shallow waters, nevertheless, harbor a diverse array of microbial communities that play a vital role in the process of biodegradation [72]. The complete transformation of polymers into MPs typically necessitates the cooperation of numerous species of microorganisms and is a complex process when mediated by biotic agents.

**Fig. 4:**
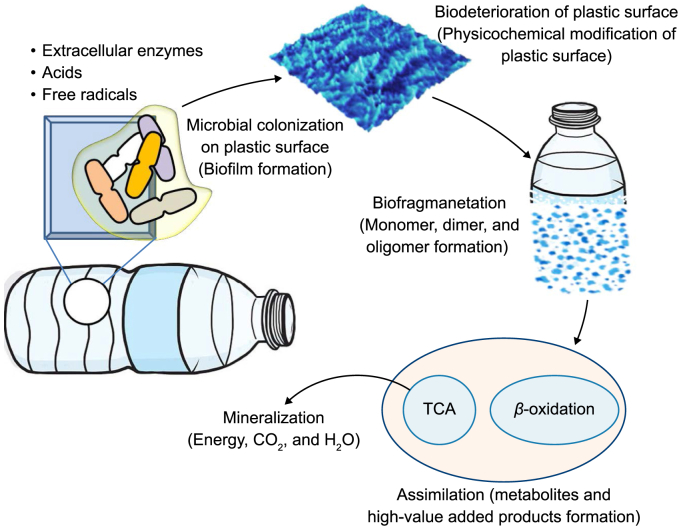
Metabolic processes involved in plastic biodegradation.

“Co-metabolism” pertains to a microbiological phenomenon in which microorganisms break down organic compounds in a communal setting, utilizing carbon and energy sources acquired from distinct substrates [73]. The availability of alternate carbon and energy sources contributes to heightened microbial enzyme activity, improving efficiency in the breakdown of substrates. A wide range of factors that affect PW's biodegradability govern the biodegradation process. These factors encompass the inherent characteristics of the polymer, such as its type, the presence of functional groups on the plastic surface, molecular weight, chemical additives, and production method. Additionally, environmental factors, including temperature, pH, oxygen concentration, and water salinity, influence PW's biodegradation process [74]. Multiple catabolic mechanisms are implicated in the biodegradation of PW (Fig. 4). Biodeterioration, characterized by changes in the surface structure and shape of the polymer, marks the initial phase of biodegradation [1]. Subsequent stages include biofragmentation, assimilation, and mineralization.

Extracellular enzymes enhance the accumulation and attachment of more contaminants onto the plastic surface, promoting microbial growth and accelerating biodeterioration [4]. This highlights the crucial function microbial extracellular enzymes and secretions perform in the biodegradation process. Fungi can degrade MPs through laccase, which accelerates the oxidative cleavage of the amorphous high-density polyethylene (HDPE) structure [75]. The oxidase enzyme aids in the breakdown of polymers, allowing for the extraction of carbonyl groups of the polymer chains. The study conducted by Sumathi et al. [76] examined the capability of the laccase-producing *Cochliobolus* sp. to degrade PVC and optimize conditions for laccase synthesis. The findings demonstrated a modification in the physical and chemical characteristics of the PVC samples, resulting in erosion and the incorporation of carbonyl groups (C–O) on the plastic material's surface.

Depolymerases and hydrolases are extracellular enzymes secreted by microorganisms (including bacteria and fungi). These enzymes can hydrolyze complex molecules into simpler structures, including monomers, dimers, and oligomers. According to the findings of Lepcha et al. [77], specific chemoorganotrophic microorganisms (*Nitrobacter* spp., *Thiobacillus* spp., *Cladosporium* sp., and *Aspergillus* sp.) are capable of producing a range of organic acids (e.g., oxalic, gluconic, glyoxylic, and citric) and other chemicals. These compounds demonstrate substantial activity during the entire phase of biodeterioration. This stage is also influenced by several other variables, such as pH, which changes due to metabolic activity within the medium [1,4]. Microbial cells can incorporate the monomers produced during the biodeterioration phase into their structure to promote their growth and development. These monomers can pass through microbial cell membranes and endure mineralization. Mineralization processes involving the monomers found within cells can generate CH_4_, CO_2_, and H_2_O in anaerobic environments [74]. On the contrary, monomers have the potential to undergo mineralization into H_2_O and CO_2_ under aerobic conditions, thereby producing energy and biomass.

**Fig. 5:**
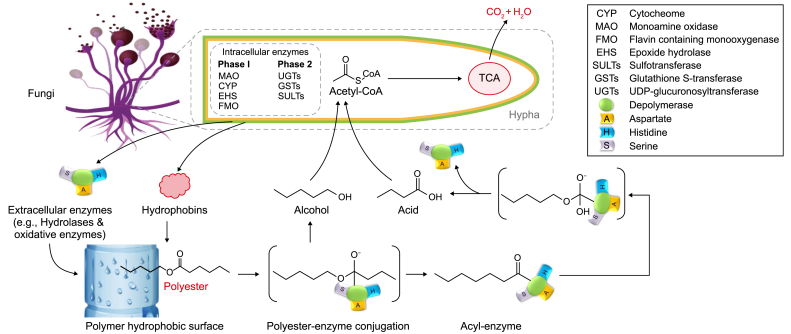
Extracellular enzymatic system-based mechanism for fungal polyester biodegradation.

The chitinase enzyme obtained from fungi, particularly *Rhizopus oryzae*, has significantly degraded PE and PET materials [78]. Fungus can cling to the plastic surface by producing proteins, including hydrophobins, cysteine-rich proteins, and polysaccharides (Fig. 5). The fungal cell can produce extracellular enzymes. Specific chemicals and enzymes enable fungi to stick to plastic surfaces and penetrate the polymeric structure [79]. The infiltration process forms pores and cavities, hence modifying the morphological properties of the plastic surface. At this stage, a physicochemical transformation reduces the polymer's resistance [11]. A yeast consortium, developed from the symbiotic microorganisms found in the termite gut, was used to degrade large molecular weight polymers. In this process, PW was used to promote yeast proliferation [12]. It is worth noting that including a supplementary carbon source inside the culture medium can potentially augment biodegradation efficiency. The impact of adding malt extract and glucose to the growth medium of *Zalerion maritimum*, a type of marine fungus, has been evaluated, and the rate of parliamentary expulsions reached 56% [80]. After 28 days, a reduction in the molecular weight of the PE-MPs was observed by *Aspergillus flavus* PEDX3 isolated from the intestine of *Galleria mellonella*, and this process was facilitated by laccase-like multicopper oxidases [81].

## 5 Formation of microplastics

Global plastic production has increased from 1.5 million tons to approximately 359 million tons over the past seven decades [82]. Projections indicate that this figure will stabilize at 500 million tons by 2025 [83]. In 2013, China dominated global plastic production, with an estimated 63 million tons of plastic manufactured in the country. Upon factoring in the plastic output of additional Asian countries, the aggregate plastic production in Asia amounts to an estimated 114 million tons [84]. The European Union was the second-largest plastic producer, producing around 50 million tons. North America made a significant contribution by producing 49 million tons of plastic. Africa, Latin America, the Middle East, and the Commonwealth produced 37 million tons of plastic [85]. MP particles substantially impact aquatic habitats as they accumulate toxic contaminants, including heavy metals and persistent organic pollutants. MPs can be produced through the degradation of larger polymers via physical, chemical, or biological factors. These MPs are known as secondary MPs [86]. However, primary MPs are intentionally added to consumer and commercial products, such as cosmetics, nappies, paints, and insecticides [87]. The chemical composition of MPs can be categorized into six groups: PE, PS, PP, PUR, PVC, and PET [88]. MPs can also be classified into five major categories: pellets, fragments, films, fibers, and foam [89].

## 6 Microplastics in aquatic systems

**Fig. 6:**
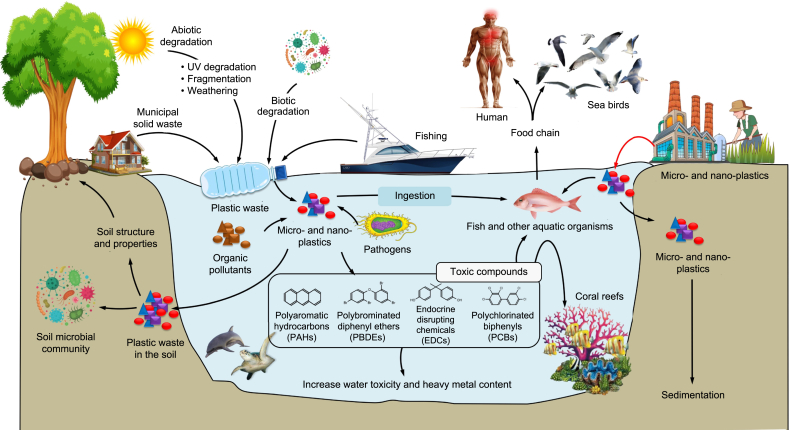
Source and life cycle of microplastics in the aquatic environment and their extended impact on humans, animals, and plants.

The distribution of MPs in aquatic ecosystems is determined by multiple factors, such as fouling and density, which ultimately determine their effects on marine animals. Following fragmentation, MPs with lower density remain buoyant on the water surface, while those with higher density sink and collect in the sediment [90]. Subsequently, MPs are exchanged between organisms, water, and sediment through processes such as ingestion, bioturbation, and excretion (Fig. 6). The introduction of freshwater, together with its associated turbulence, can lead to the dispersion or disruption of MPs in the aquatic environment. A recent study using modeling techniques has revealed that the movement of MPs in rivers is strongly influenced by the flow of water, which affects how they are transported into aquatic ecosystems [91]. The impact of MPs is more substantial compared to the original larger plastic particles due to their capacity to infiltrate living tissue. Therefore, it is imperative to investigate the impact of these substances on the aquatic ecosystem and its organisms.

The presence of MPs in drinking water, without an effective purification or treatment system to address these pollutants, directly impacts human health. This impact manifests in various diseases, such as cancer, genetic mutations, inflammations, and other related ailments [1,4]. Both primary and secondary MPs are capable of invading marine environments. Occasionally, macro- and mega-plastic particles have the potential to be discharged into the environment, subsequently undergoing degradation and transforming into MPs and NPs (Fig. 6). Ultraviolet radiation is the primary mechanism responsible for the degradation of plastics. The photo-degradation leads to the deterioration of the structural integrity of high molecular weight polymers through oxidative reactions that affect the polymeric structure [92].

While numerous studies have brought attention to MPs in aquatic organisms, there remains a dearth of research regarding the consequences of ingestion by different species. In aquatic ecosystems, MPs have the potential to be consumed by invertebrates, fishes, birds, and marine mammals (Fig. 6). Toxic substances are incorporated into MPs through ingestion; these substances are either adsorbed onto the surfaces of MPs during transportation or added as additives during the manufacturing process [93]. Anticipated quantities of MPs are to be found in aquatic biota; nevertheless, experimental data is inconclusive as to whether MPs convey higher concentrations of pollutants to tissues compared to sediments or if they can transfer sufficient concentrations of pollutants to impair organism functions [94].

Pollution with MPs has been identified as an issue in freshwater and marine ecosystems [95]. The prevalence of MPs in freshwater across Europe, Africa, Asia, and North America has been reported [[96], [97], [98]]. Freshwater and marine systems have similarities in the forces that facilitate the transport of MPs and their potential effects on organisms. Nevertheless, a notable distinction between freshwater and marine ecosystems is that MPs are more closely associated with their primary materials in freshwater. Consequently, variations in the composition of MPs have emerged in freshwater ecosystems such as rivers, exhibiting a discernible pattern in size, shape, and relative prevalence [99]. Despite reports of MPs being detected in freshwater, data regarding their presence and distribution in freshwater ecosystems are scarce. Since humans depend highly on freshwater sources for drinking and other purposes, the lack of knowledge regarding the potential health effects of MPs transferred from freshwater to terrestrial environments is alarming.

### 6.1 Life cycle of microplastics in aquatic systems

Bioaccumulation is a part of the MPs' life cycle (Fig. 6). The aforementioned process typically commences with the discharge of primary or secondary MPs into terrestrial and aquatic ecosystems, followed by their conveyance into water systems. As a result, MPs enter the food chain of aquatic organisms and accumulate in their tissues through bioaccumulation. As zooplankton, small fish, larger fish, and other organisms ingest them, the MPs progressively ascend the trophic levels. Ingestion of these contaminants has been demonstrated to induce detrimental physiological responses in aquatic organisms, such as sea turtles, mussels, and fish. Such effects may manifest as compromised digestive and immune systems, ultimately culminating in their demise [100,101]. MPs may adversely affect human health, given that their entry into the food chain can occur via ingesting contaminated fish or other aquatic organisms. Human brain cells are susceptible to cytotoxic effects from MPs [102]. MPs can absorb antibiotics and other hazardous compounds due to their large surface area. This worsens the issue of MP contamination [103]. Moreover, the environmental cycle of MPs persists through their potential excretion by humans or their discharge as PW products. Recent studies have conducted surveillance of MPs in drinking and mineral water bottles [104,105]. Developing novel approaches and cutting-edge methodologies to eradicate plastics from water sources is crucial, as conventional methods are inadequate in eradicating MPs owing to their diminutive dimensions. Consequently, there has been a rise in the prevalence and persistence of MPs in the environment [106].

### 6.2 Adverse effects of microplastics

**Fig. 7:**
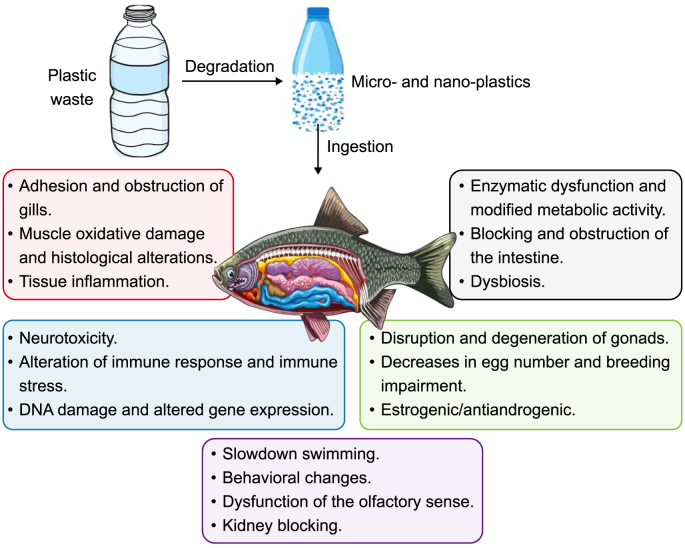
Influence of microplastics on fish.

The environment and living organisms, including humans, have been adversely affected by MPs. The toxicity of MPs poses an ongoing and persistent risk to species within the environment. Table 3 illustrates the adverse effects of MPs on aquatic organisms [[107], [108], [109], [110], [111], [112], [113], [114], [115], [116]]. MPs have been found to induce reproductive failure in mammals and aquatic organisms and can potentially lead to mortality if the dosage surpasses a certain threshold [117]. Exposure to MP leads to a decrease in both the hatching rate and larval length of the eggs [118]. Administering MP can induce abnormal animal behavior by blocking the gastrointestinal tract [119]. The study by Huang et al. [120] investigated the influence of MPs on red tilapia. MPs were found to exhibit a higher degree of accumulation in fish tissue, particularly in the liver, compared to other tissues, as illustrated in Fig. 7. MPs proliferate and disseminate throughout the gastrointestinal tract, muscular system, and gills of fish. Evidence links marine animal ingestion of MPs to oxidative stress, cytotoxicity, alterations in the physiology of the gastrointestinal tract, growth inhibition, depression in the immune system, and differential gene expression [121]. MPs can induce adverse modifications in the gastrointestinal tract physiology of marine organisms. For instance, adult zebrafish may experience an imbalance of gut microbiota, enterocyte splitting, and villi fracture [122]. Zebrafish exposed to MPs for 21 days demonstrated microbiota dysbiosis, a condition characterized by an alteration in the regular metabolic process [123].

**Table 3:** Adverse effects of microplastics on aquatic organisms.

Organism	Toxic impact of microplastics (MPs)	Reference
European sea bass (*Dicentrarchus labrax* L.)	• Alterations of the intestine and liver. • Reduce antioxidant activity and increase oxidative stress. • PVC-MPs enhance the respiratory burst and phagocytic activities of head kidney leucocytes. • PE-MPs increase skin mucus immunoglobulin M level.	[107]
Zooplankton (*Daphnia magna*) and Crucian carp (*Carassius carassius*)	Cholesterol and triglycerides alteration due to the presence of MPs in blood and tissue.	[108]
Sea turtle	Increase mortality.	[109]
Zebrafish	Inhibit the expression of genes related to cardiac development.	[110]
Cetacean species	• Acute and chronic injuries. • Increase mortality.	[111]
Baleen whales	Dysfunction of digestive processes.	[112]
Oyster	• Reduce sperm velocity. • Decrease oocyte number.	[112]
European sea bass	Blockage of the intestinal lumen.	[112]
Nematode	Intestinal damage.	[112]
Lobster	Reduce larval survival rate and metamorphosis.	[113]
*Dunaliella tertiolecta*, *Chlorella vulgaris,* and *Thalassiosira pseudonana*	A reduction in photosynthesis due to the presence of PE-MPs.	[114]
*Chlamydomonas reinhardtii*	PE- and PP-MPs enhanced molecular toxicity of *C. reinhardtii* and enhanced hetero aggregates.	[115]
Zebrafish	Increase metallothionein transcript in larvae.	[116]

Abbreviations: MPs, microplastics; PVC, polyvinyl chloride; PE, polyethylene; PP, polypropylene.

The toxic effects of MPs on marine organisms have been the subject of numerous studies [25,124]. Furthermore, research has examined the accumulation of MPs in clinical environments from human sources, such as meconium, stool, colectomy samples, and human placenta [125]. It has been documented that aquatic mammals consume a wide range of polymers, such as PE, PP, polyester, and nylon [121]. Additionally, MPs exert substantial toxicity on animals via various routes of exposure, including dermal, subcutaneous, intraperitoneal, oral, and intravenous. Introducing MPs through either a direct or indirect pathway can lead to many repercussions. Direct exposure refers to the situation where contaminants directly touch an organism [126]. This usually leads to acute toxicity, which lasts for a short duration. Chronic organ toxicity occurs when MPs become entangled in the food web, leading to indirect exposure. MPs induce detrimental consequences in fish, vertebrates, and coral and sea urchins. Scleractinia coral *Pocillopora damicornis* exhibited an activated stress response to acute MPs exposure. Simultaneously, its immune system and detoxification processes were inhibited via the extracellular signal-regulated kinases and *c*-Jun *N*-terminal kinases signaling pathways [127]. In the interim, larval development was impeded and lengthened in sea urchins due to MP exposure; the extent of this impact was proportional to the dose of exposure [128]. In addition, Qiao et al. [123] found that zebrafish exposed to MPs suffered from oxidative stress due to alterations in glutathione levels and elevated amounts of superoxide dismutase in their intestinal tissues. Overall, these studies demonstrate the far-reaching adverse effects that MPs can inflict on various marine organisms. Invertebrate growth is inhibited and slowed by the presence of MPs. Deng et al. [129] report that MPs may inhibit the development of *Skeletonema costatum* and impose growth restrictions on *Chlorella pyrenoidosa* and *Tetraselmis chuii*, both freshwater algae. Moreover, Jaikumar et al. [130] establish that reproductive toxicity in Daphnia could be induced by protracted MP exposure.

## 7 Recent strategies for microplastic remediation

Physical, chemical, and bioremediation technologies can be used to clean up contaminated areas. There has been a growing interest in employing advanced techniques for efficient MP removal in recent years. These techniques include membrane bioreactor (MBR), synthetic biology, organosilane-based techniques, biofilm-mediated MP remediation, and nanomaterial-enabled strategies.

### 7.1 Biofilm-mediated MP remediation

The surface of MPs is rapidly colonized by microorganisms, which establish a durable biofilm upon their introduction into an aquatic environment [131]. In biofilms, specific bacteria can decompose organic contaminants and promote the adhesion of pollutants via MPs [132]. Nevertheless, a more significant aspect is that the interaction between biofilms and MPs could potentially induce alterations in the polymer surface's chemical and physical properties, thereby leading to the MPs' biological degradation. Table 4 depicts the biofilm-forming bacteria for MP biodegradation [80,[133], [134], [135], [136], [137], [138]]. It has been demonstrated that *Rhodococcus ruber* can produce biofilms and colonize PE surfaces [139]. The average molecular weight of the PE samples experienced respective reductions of 14% and 21% [139]. Thus, a study was performed to determine whether the development of biofilms could alter the physicochemical properties of MPs [140]. MPs coated with biofilms experience substantial surface degradation in high concentrations of methane gas. This may be a factor in the escalation of bacterial aggregation [141]. The degradation of MPs yields solely CO_2_ and H_2_O as metabolites, neither of which are detrimental to the ecosystem [142]. Substantial degradation of MPs treated with biofilms can result from the formation of bacterial aggregation, which is facilitated by the presence of an abundance of methane gas in an environment [141].

**Table 4:** Biofilm-forming bacteria for the biodegradation of microplastics.

Plastic type	Bacteria	Reference
PE	*Rhodococcus ruber*	[80]
PET	*Bacillus muralis*	[133]
PE	*Bacillus subtilis*	[134]
PVC	*Mycobacterium* sp. NK0301	[135]
PVC	*Microbacterium* sp. NA23	[135]
PP	*Rhodococcus* sp.	[136]
PP	*Bacillus* sp.	[136]
PS	*Pseudomonas aeruginosa* NB26	[137]
PP	*Stenotrophomonas panacihumi*	[138]
PS	*Bacillus* sp. NB6	[137]

Abbreviations: PE, polyethylene; PP, polypropylene; PET, polyethylene terephthalate; PVC, polyvinyl chloride; PS, polystyrene.

Biofilms are abundant and easily accessible in their natural habitat [143]. Utilizing glucose as an exogenous carbon source accelerates the degradation of MPs compared to natural biofilms [144]. Conversely, biofilms can promote the attachment of MPs to environmental pollutants, serving as transporters and intensifying the environmental risk of MPs in the ecosystem [88]. Upon introduction of MPs into a water-based environment, bacteria promptly inhabit their surfaces and generate biofilms that promote the adsorption of MPs to pollutants present in the environment [145]. Nevertheless, if the biofilm is cultivated and formed in advance under controlled conditions before being exposed to MPs, the issue can be significantly mitigated.

Integrating the biofilm degradation technology of MPs into the treatment of MPs in freshwater sources or their *in situ* cleanup can absorb more environmental pollutants while MPs degrade in the aqueous environment [146]. The growth of biofilms on MP surfaces is influenced by many factors, including pH, salinity, temperature, and UV radiation. It has been found that a maximum deterioration of 20% could occur [141]. The principal factor contributing to this occurrence is the duration necessary for microorganisms to modify the inherent properties of MPs. Although biofilms are a potential approach for degrading MPs, the current extent of degradation is inadequate. MPs undergo degradation by biofilm-producing bacteria due to the inability of these organisms to directly use macro-polymers. Upon the entry of MPs into the biofilm, several extracellular oxidases and hydrolases degrade the large macromolecular polymers into smaller oligomers and monomers. Subsequently, the bacteria in the biofilm uptake and commence the conversion of these shorter-chain polymers [6]. Ultimately, MPs can undergo mineralization in the presence of bacteria, resulting in the formation of CO_2_ and H_2_O.

Eliminating MPs using the biofilm approach typically involves four steps [146]. Initially, bacteria gather on the surface of MPs and alter their composition. Then, the bacteria break down the additives and monomers present in the MPs. Subsequently, enzymes or free radicals the bacteria produce attack the MPs and their additives, causing them to deteriorate and become mechanically unstable. Finally, bacteria destroy the MPs by disintegrating microbial filaments and water infiltration into the polymer matrix. It is believed that the most crucial stage of degradation occurs during the second step [146].

Many additives are commonly incorporated into plastic products to improve or alter their chemical and mechanical properties. The conversion of plastic waste into MPs leaves behind these compounds, and their existence substantially impedes the degradation process of MPs. Before the subsequent degradation process, the additives must be leached from the MPs' interiors to eliminate them. The degradation of polymer additives by microorganisms facilitates the formation of biofilms and initial bacterial adhesion to the particles' exterior surface [147]. This process promotes the formation of biofilms, which facilitates the degradation of MPs and additives by the biofilms. The complexity of MP decomposition can be reduced by identifying and cultivating microorganisms that substantially influence the process and subsequently employing them to achieve optimal outcomes.

### 7.2 Synthetic biology and organosilane-based techniques

Synthetic biology has been routinely used to study the interactions between microbes and the natural environment, specifically focusing on the degradation of polymers. This inquiry is frequently carried out using “omic” methodologies [148,149]. Another essential aspect in synthetic biology is the development of a metabolic pathway designed to enhance the degradation of fossil-based petroleum waste [150]. Nevertheless, unresolved inquiries remain about the diverse array of bacteria that facilitate the degradation of synthetic polymers and the enzymes accountable for this process. Hence, future studies must prioritize the investigation of techniques aimed at describing and discerning a resilient microorganism capable of degrading plastic, along with comprehending its enzymatic process. To overcome the obstacles encountered in the biodegradation of MPs, it is imperative to do extensive research in environmental microbiology, biotechnology, gene engineering, and protein engineering. Combinatorial approaches encompassing metabolic engineering, bioinformatics, molecular biology, genetics, and systems biology hold the potential to yield groundbreaking insights into the field of plastic biodegradation in the near future.

The innovative method utilizing organosilane, which integrates a physical agglomeration process with a water-induced chemical fixing phase, yields robust particle formation and enduring agglomerates [151]. Consequently, the organic groups can be modified to accommodate a wide range of surface chemistries and polymer varieties. Modifying reactive and organic groups makes it possible to customize organosilanes' reactivity to suit different water compositions [152]. The considerable versatility and novelty of organosilanes render this relatively new and understudied technique highly promising for water treatment and MP elimination applications.

### 7.3 Membrane bioreactor (MBR)

**Fig. 8:**
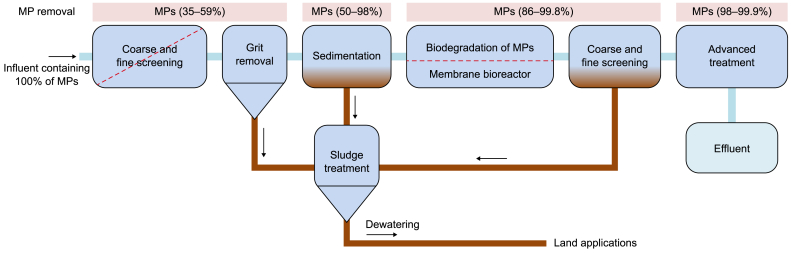
Bioreactor technology for the elimination of microplastics.

The implementation of MBR technology has demonstrated significant effectiveness in eliminating more than 90% of MPs from wastewater, as opposed to conventional methods used in WWT plants [153]. However, it is important to note that implementing these filtration modules can be costly and prone to rapid clogging. The utilization of bioreactor technology for the elimination of MPs yields a high degree of purity due to the prior removal of most contaminants during previous treatment stages (Fig. 8). Consequently, there is a possibility of reevaluating these MPs through plastic recovery. Recent developments in membrane technology, particularly in the field of nano-filtration membranes, have led to the emergence of reverse osmosis and ultrafiltration hybrids [154]. These hybrid systems show great potential in effectively reducing MPs while avoiding issues such as clogging [153].

MBR is a complex system that integrates a biological catalyst, such as bacteria or enzymes, with a partition approach and operates through a film-based mechanism [148]. MBRs have been used to mitigate the contamination of water-containing MPs. The degradation and removal efficiency of an MBR are greatly influenced by the physicochemical features of pollutants and operational factors such as feeding rate, hydraulic retention period, and concentration [155]. The capacity of *Ideonella sakaiensis* to effectively metabolize PET as a carbon source is a noteworthy advancement with potential near-term implications in the field of MBRs [156]. The aforementioned bacterial species possessed distinct enzymes that effectively catalyzed the conversion of PET into harmless organic compounds, namely terephthalate and ethylene glycol. The involvement of intricate enzymatic pathways in the size reduction of MPs was identified through a comprehensive examination of *Euphrasia superba* [157]. The incorporation of these enzymes into the MBR is anticipated to occur in the near future, facilitating the biodegradation of PET-MPs. Therefore, the utilization of an MBR has the potential to serve as a viable method for the bioremediation of polluted water.

### 7.4 Nanomaterial-enabled strategies for microplastic remediation

The advancements in nanomaterial research have substantially reinforced the significance of utilizing nanomaterials and their beneficial effects in WWT. Nanomaterials make energy-efficient remediation processes possible. Numerous novel photocatalyst structures have been developed to enhance the light absorption response of the material by limiting charge-carrier recombination and increasing the number of light-active sites [158,159]. To attain this objective, it is frequently practiced to design the photocatalyst at the nanoscale or to dope the base photocatalyst with an extensive variety of functional nanomaterials [160,161]. By optimizing the light absorption response, it becomes feasible to induce photocatalytic activity using visible light rather than the exceedingly intense UV light. This facilitates the implementation of photocatalytic processes powered by solar energy. Using nanomaterials for magnetism separation has been documented as a straightforward and economical method to eliminate MPs [162]. Thus, the separation of magnetism can be achieved through the development of a nanomaterial capable of interacting with the MP's surface. Magnetized MPs of small size can be extracted from an aqueous medium using the magnetic recovery method. The magnetization of PE-, PP-, PS-, and PET-MPs of diverse sizes and shapes was achieved by utilizing magnetite (Fe_3_O_4_) nanoparticles [163]. This process facilitated the removal of the MPs from various water sources, including ultrapure water, artificial seawater, and actual water collected from rivers and sewage. The process of MPs adsorbing onto one another has been observed, leading to the determination of the optimal concentration of Fe_3_O_4_ nanoparticles as 1.3 g L^−1^ [162]. The efficacy of MP elimination exhibited variability in response to their dimensions, morphologies, and the aqueous medium. While external forces can remove all types of magnetized MP, the PET removal rate was notably lower compared to PE, PP, and PS [164]. This can be attributed to the comparatively feeble hydrophobic nature of PET. The electrostatic attraction between positively charged Fe_3_O_4_ nanoparticles and negatively charged MPs in seawater increased the MPs' adsorption and magnetism, thereby increasing the overall removal efficacy. The established methodology was implemented to eliminate MPs, primarily PE and PES, identified in river water, domestic effluent, and actual seawater [165]. The respective removal rates for MPs were 81.3%, 82.3%, and 80.6% [162].

Table 5 summarizes the performances of nanomaterials in MP removal [[166], [167], [168], [169], [170], [171], [172]]. Nanomaterials have been predominantly implemented in adsorption and photocatalysis, where their distinctive characteristics are leveraged to surmount the drawbacks associated with larger materials [173]. For adsorption and photocatalysis, one of the most desirable qualities exhibited by these nanomaterials is their substantial surface area. Increasing the uptake capacity makes it possible to conduct treatment procedures on a larger scale before the materials reach their saturation point. Hybrid nanomaterials with top-down and bottom-up hierarchical designs are feasible due to the distinctive structural properties of nanomaterials [174]. These designs can generate favorable synergistic effects that enhance the efficacy of MP removal, in contrast to bulk nanomaterials that are typically employed as a singular entity. Although growing interest has been in treating MPs in aqueous matrices, the application of nano-enabled materials in this regard is still in its nascent phase [175,176]. An increasing number of research endeavors are currently devoted to investigating the potential of nanomaterials or nano-functionalized materials as substitutes for traditional materials. However, their primary emphasis remains on advancing nanostructured adsorbents and photocatalysts.

**Table 5:** Removal of microplastics using nanomaterials.

Microplastics	Nanomaterials	Microplastic concentration	Process	Removal efficiency (%)	Reference
PS	TiO_2_	5 μm	Catalysis	100	[166]
PS	3D rGO	0.25 ppm	Adsorption	73	[167]
PE, PS	Fe-HDTMS	10–20 μm	Magnetism adsorption	92	[168]
PS	UiO-66-OH	1 ppm	Adsorption	86	[169]
PE, PET	Magnetic CNTs	48 μm	Magnetism adsorption	100	[170]
PS	GO, *O*–C_3_N_4_	1 ppm	Adsorption	91	[171]
Cosmetic product-extracted microplastic	Mn@NCNTs	5000 ppm	Catalysis	50	[172]

Abbreviations: PE, polyethylene; PP, polypropylene; PET, polyethylene terephthalate; PS, polystyrene; TiO_2_, titanium dioxide; 3D rGO, three dimension reduced graphene oxide; Fe-HDTMS, carbon steel-hexadecyltrimethoxysilane; UiO-66-OH, zirconium-based metal-organic framework; CNTs, carbon nanotubes; GO, graphene oxide; *O*–C_3_N_4_, oxygen-doped carbon nitride; Mn@NCNTs, manganese carbide nanoparticles.

It is noteworthy that, notwithstanding the widely acknowledged capabilities of membrane filtration in eliminating MPs, inadequate emphasis has been placed on the advancement of high-performance membranes — particularly those facilitated by nanomaterials — to augment the throughput of the process or resolve concerns related to membrane fouling. The photocatalytic membrane is an emerging class of membrane that combines the physical separation of membrane process and chemical degradation of photocatalyst to enable more effective WWT [177]. This approach offers an appealing way to merge two MP treatment methods, which operate on the principles of capture and degradation within a single entity. Furthermore, photocatalytic membranes can effectively address the secondary pollution of MPs via membrane [178]. The photocatalysts that are incorporated into the outermost layer of the photocatalytic membranes are capable of degrading MPs. Photocatalytic membranes have proven effective in treating diverse wastewater pollutants, such as emerging chemicals and micropollutants [154,179]. The favorable results documented in these investigations offer a strong indication of the potential of photocatalytic membranes in the treatment of MPs.

## 8 Conclusions and future perspectives

Plastics are one of the most crucial breakthroughs in global industry. Over time, the discharge of MPs from diverse origins into aquatic ecosystems has become an increasingly significant peril for living organisms and the environment. Given the numerous detrimental impacts that MPs introduce into the environment, specifically in aquatic ecosystems, developing effective approaches for mitigating their prevalence is critical. Water treatments are critical engineering tools that can provide immediate solutions to control, mitigate, or even eliminate MP pollution, whereas preventative measures are the most effective approach to MP remediation. Although there is limited evidence on the health risks posed by MPs to humans, significant evidence supports the ecological risks presented by MPs to certain aquatic and terrestrial organisms. MPs could be remediated using various physical, chemical, and biological techniques. Microorganisms, including bacteria, fungi, and algae, as well as specific enzymes, sophisticated molecular techniques, and biomembrane treatment, can engage in MP remediation strategies. Nonetheless, there is a pressing need to advance these technologies even further. Nanomaterials encounter numerous obstacles when attempting to address the shortcomings of conventional methods employed in water treatment processes. Notably, scaling up to large-scale treatment and ensuring reproducibility pose particular challenges.

This study emphasizes several factors by integrating the diverse expertise in the field regarding the remediation of MPs in aquatic ecosystems. There are numerous research gaps and issues pertaining to MPs that necessitate additional investigation and exploration in future studies.

• Additional investigation is needed to address knowledge gaps about the environmental health risks posed by MPs.
• Effective management strategies and stringent laws are essential for addressing the issue of MPs.
• To optimize efficacy and minimize adverse effects, it is imperative to integrate MP treatment technologies and strive to enhance the quality and performance of plastic alternatives, including bioplastics.
• Strict regulations must be applied following the triple R principle — Reduce, Reuse, and Recycle — to reimagine the utilization of PW and mitigate the adverse effects of MP's pollution.
• Novel enterprises have the potential to contribute to the development of a circular economy for plastics through the establishment of material recovery facilities, promotion of domestic waste segregation, and facilitation of community integration for workers engaged in the informal waste-picking industry.
• Developing advanced treatment technologies is essential for enhancing the removal efficiency of MPs in drinking water and WWT plants.
• Further research is also needed to understand the toxic impacts of MPs on humans and animals and develop suitable alternatives for single-use face masks and PW generated by the medical industry.
• Further research is still required to assure optimal compatibility between nanomaterials, remediation approaches, and various forms of MPs.
• Further research is also needed to explore methods of recycling and reusing MPs in the environment while adhering to low carbon limitations.
• When formulating a plan to mitigate plastic consumption, various elements must be considered, including infrastructure, economic circumstances, the nature of MPs discharged, and alternative options to shift towards an economy less reliant on plastic.

## CRediT authorship contribution statement

**Sameh S. Ali:** Conceptualization, Data Curation, Formal Analysis, Software, Visualization, Writing - Original Draft, Writing - Review & Editing. **Tamer Elsamahy:** Methodology, Writing - Original Draft. **Rania Al-Tohamy:** Formal Analysis, Methodology, Writing - Original Draft, Writing - Review & Editing. **Jianzhong Sun:** Conceptualization, Data Curation, Investigation, Validation, Writing - Review & Editing.

## Declaration of competing interest

The authors declare that they have no known competing financial interests or personal relationships that could have appeared to influence the work reported in this paper.
